# 749 Does a Dedicated Burn Therapist Add Value in an Outpatient Setting?

**DOI:** 10.1093/jbcr/irad045.224

**Published:** 2023-05-15

**Authors:** Kristin Rainey, Jennifer Rosenthal

**Affiliations:** Medical City Plano, Plano, Texas; Medical City Plano, Plano, Texas; Medical City Plano, Plano, Texas; Medical City Plano, Plano, Texas

## Abstract

**Introduction:**

Caring for burn patients requires specific knowledge, skill, and a collaborative team approach from multiple disciplines including occupational therapy. In the hospital system, therapists are often under direction of rehabilitative services, not nursing administration. There are specific productivity expectations within the rehabilitation department which rely heavily on time-based billed interventions. This hospital had an occupational therapist (OT) assigned to the burn clinic as well as acute care patients to help improve the therapist’s productivity. With a growing burn clinic, some patients were missed by the OT while they were providing services elsewhere in the hospital. With support and advocacy from the nursing Director of burn services, a full-time occupational therapist was hired solely for the outpatient burn clinic to improve the care of burn patients. Prior research has focused on the importance of OT in treatment of the burn patient, however no research was found in regard to the benefit of hiring a therapist specifically for an outpatient burn setting. This information is crucial for hospital administration to determine the need of having a burn-dedicated therapist. Specialized training in wound care, splinting, measuring and assessing for custom compression garments, and burn-specific evidence-based practice is required for a therapist to be competent in a burn center. Having a specialized burn therapist available is vital for the optimal care of the burn outpatient. This study analyzes the benefits of having an OT dedicated to the burn clinic compared to an OT being staffed for both the inpatient and outpatient needs.

**Methods:**

A retrospective chart review was performed for 6 months prior to hiring a full time OT in the clinic to 6 months after.

**Results:**

Preliminary data taken from October 2021 to present indicate a growth in OT services provided in the burn clinic from an average of 0.64 patients and 2.44 billed productive units per day to 3.9 patients and 12.4 units per day. At over 500% increase in just 3 months of hiring a full time OT, the data indicates that adding a burn therapist in the clinic increases number of patients seen. Productive units billed by the therapist also increased over 400%. Data regarding the amount of revenue are pending.

**Conclusions:**

Having a dedicated burn therapist in an outpatient clinic increases the number of patients receiving necessary OT services and subsequently improves financial reimbursement.

**Applicability of Research to Practice:**

Consistent availability of the OT, increased understanding of the role, purpose, and value of OT for the burn patient, increased communication with providers, and higher efficiency due to proximity to patients and providers result in increased use of OT services. Hiring a dedicated burn therapist in an outpatient setting should be considered by burn centers to increase number of patients seen and increase productive billable units by OT within the burn clinic.

